# Robotic-assisted extraction of giant gastric trichobezoar: a case report

**DOI:** 10.1093/jscr/rjac472

**Published:** 2022-10-21

**Authors:** Eduardo Serpa, Emmanuel Luciano, Felipe Pacheco, Maher Ghanem

**Affiliations:** Department of Surgery, Central Michigan University College of Medicine, Saginaw, MI, USA; Department of Surgery, Central Michigan University College of Medicine, Saginaw, MI, USA; Department of Surgery, Central Michigan University College of Medicine, Saginaw, MI, USA; Department of Surgery, Central Michigan University College of Medicine, Saginaw, MI, USA

## Abstract

Trichobezoar is a rare condition, almost exclusively seen in young females with certain psychiatric disorders. Trichobezoars are usually confined within the stomach and the complications include ulceration, perforation, intussusception and obstruction for which surgery is usually required. Most of the reported cases of giant gastric trichobezoar extraction underwent an exploratory laparotomy with only a few reported cases that underwent a successful laparoscopic approach. This case report details the surgical management of the first case of a giant obstructing gastric trichobezoar extraction using robotic-assisted surgery.

## INTRODUCTION

A bezoar is an indigestible conglomeration trapped in the gastrointestinal tract that was intentionally or accidentally ingested [1]. There are different types of bezoars according to the composition which include trichobezoar (ingested hair), phytobezoar (plant-based material), pharmacobeozar (medications) and lactobezoar (milk protein) [1].

Trichobezoar is a rare condition, almost exclusively seen in young females and the risk factors include psychiatric disorders like trichophagia, obsessive compulsive disorder and anorexia nervosa [1, 2]. Trichobezoars are usually confined within the stomach and the complications include ulceration, perforation, intussusception and obstruction [2]. Symptomatic trichobezoar are managed with a combination of endoscopic and operative techniques. This case report details the first case of a giant gastric trichobezoar extraction utilizing robotic-assisted surgery.

## CASE REPORT

**Figure 1:**
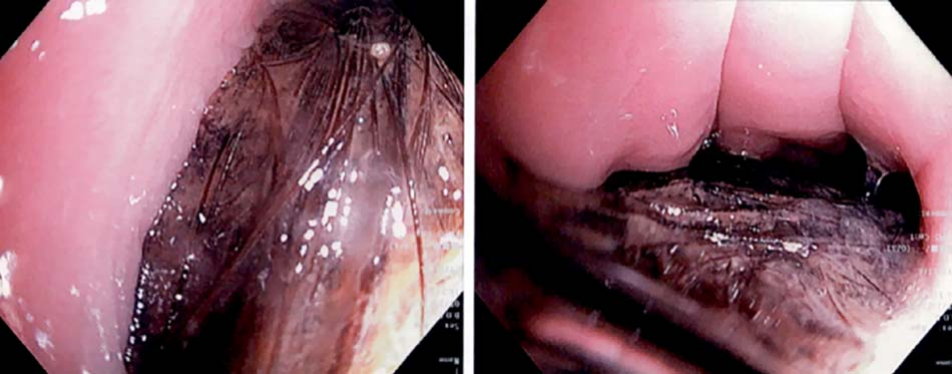
EGD with presence of trichobezoar in stomach.

A 16-year-old female with a past medical history of Tourette syndrome presented to the gastroenterology service with a 9-month history of colicky upper abdominal pain associated with nausea and occasional emesis. The patient reported decreased appetite with unintentional weight loss (10 pounds over the last 2 months). The patient underwent an esophagogastroduodenoscopy (EGD) which revealed a large, impacted gastric trichobezoar, not amenable to endoscopic treatment (Fig. 1). Surgical consultation was obtained and after extensive discussion with the family, the decision to proceed with operative removal of the trichobezoar utilizing a minimally invasive robotic approach was made.

**Figure 2:**
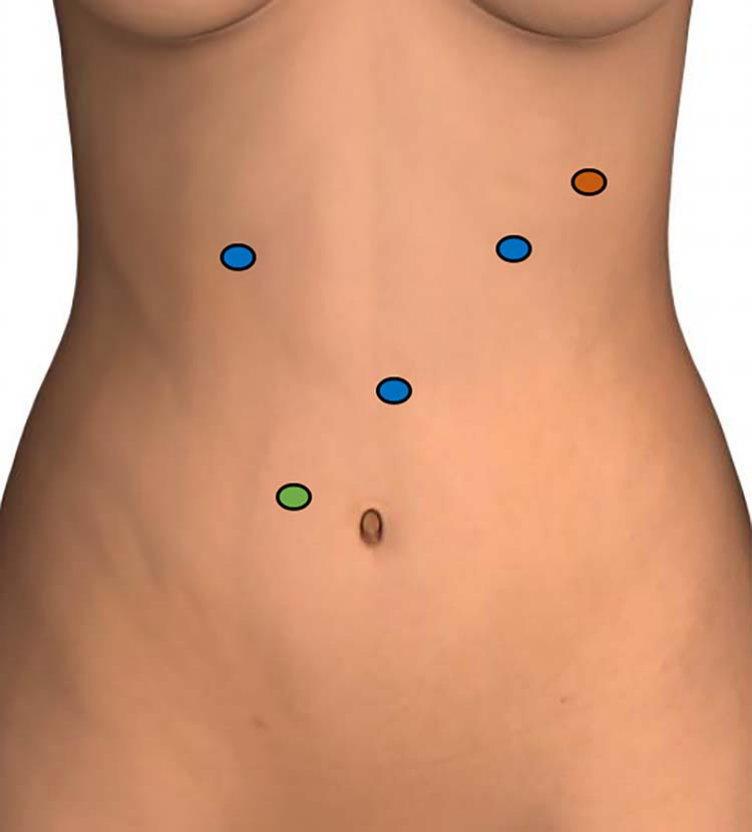
Port placement; blue circles: 8-mm robotic ports; green circle: 12-mm assistant port; red circle: 5-mm assistant port.

**Figure 3:**
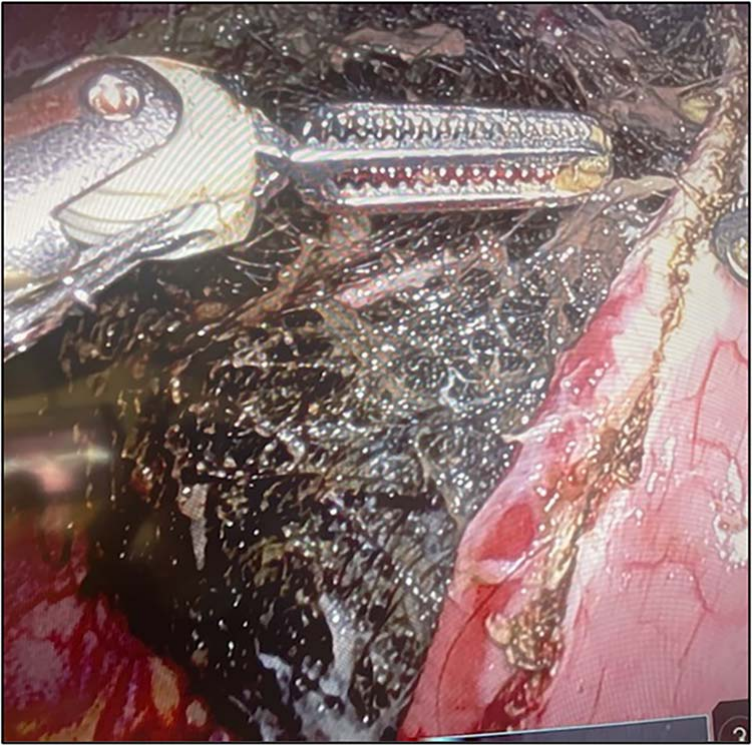
Gastrostomy with removal of trichobezoar.

**Figure 4:**
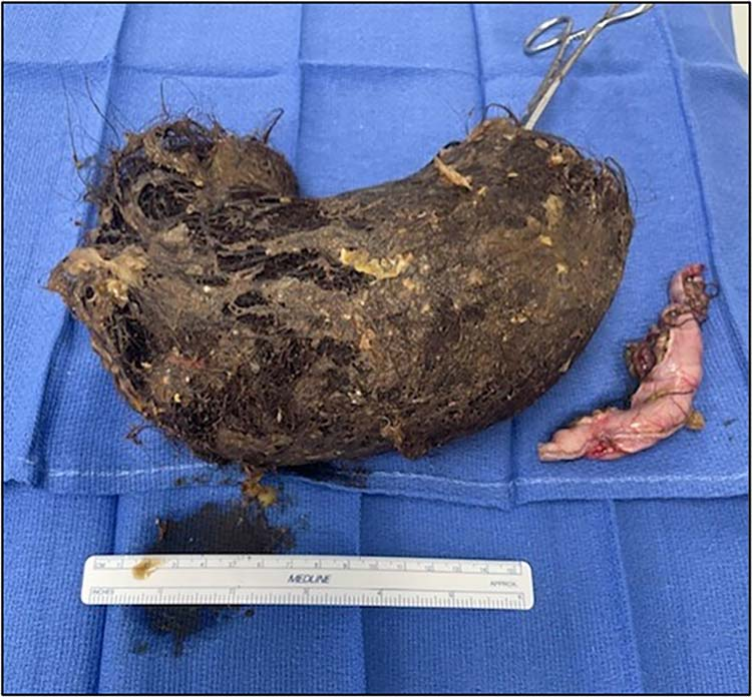
Trichobezoar.

The patient was taken to the operative room and after adequate general anesthesia was induced, pneumoperitoneum was established using the Veress needle technique. Five robotic ports were placed under direct visualization (Fig. 2). The daVinci Xi surgical platform was docked. An 8-cm longitudinal gastrotomy from the pylorus to the mid-gastric body was created using hook electrocautery. Using gentle traction, the large trichobezoar was extracted, following the curvature of the stomach (Fig. 3). Multiple silk stay-sutures were placed to ensure proper alignment of the gastrotomy. The gastrotomy was closed using a linear gastrointestinal anastomosis stapler. The staple line was reinforced with 2-0 V-loc absorbable suture in a running fashion. A 4-cm supraumbilical midline incision was made for extraction. An Alexis wound protector was placed and the specimen was removed from the peritoneal cavity (Fig. 4). There were no complications post-operatively. Diet was slowly advanced and the patient was discharged home on postoperative Day 4.

## DISCUSSION

Trichobezoars are uncommon and almost exclusively seen in young females with psychiatric disorders, such as in our case [1–3]. There are different treatment modalities for trichobezoar. Enzymatic degradation has been reported to be ineffective in trichobezoar [4]. Endoscopic removal is an option for small trichobezoars. Reports of successful endoscopic removal of trichobezoars are scarce, however, mostly outnumbered by reports documenting failed attempts at endoscopic removal [2, 5].

The vast majority of cases of trichobezoar reported in the literature have been treated through midline laparotomy [2–4]. Nirasawa *et al*. [6] reported the first case of laparoscopic removal of a large gastric trichobezoar. For large trichobezoars, a disadvantage of minimally invasive surgery is the need for an additional incision for specimen extraction. Zmudzinski *et al*. [7] reported a successful laparoscopic removal of a large trichobezoar using a Pfannenstiel incision for specimen extraction. Sharma *et al*. [8] also reported a laparoscopic removal of a large gastric trichobezoar in which the specimen was placed in an endo-bag, and it was removed through a 4-cm supraumbilical midline incision. In our case, we elected to perform a small 4-cm supraumbilical incision for extraction and we used an Alexis wound protector to decrease the risk of surgical site infection. To our knowledge, this is the first case of gastric trichobezoar removal using robotic-assisted surgery. We believe that robotic-assisted minimally invasive surgery is an adequate approach for gastric trichobezoars extraction and is at least equivalent to the laparoscopic approach.
